# The complex involvement of the digestive tract in human defense behavior – structural and functional arguments

**DOI:** 10.25122/jml-2022-0096

**Published:** 2022-09

**Authors:** Ilinca Savulescu-Fiedler, Adriana Luminita Gurghean, Roxana-Nicoleta Siliste

**Affiliations:** 1 Internal Medicine and Cardiology Department, Coltea Clinical Hospital, Bucharest, Romania; 2 Department 1 Medical Semiology, Faculty of Medicine, University of Medicine and Pharmacy Carol Davila, Bucharest, Romania

**Keywords:** gut-brain interaction, enteric nervous system, enteroendocrine cells, autonomic nervous system, ATP – adenosine triphosphate, CCK – cholecystokinin, CNS – central nervous system, CO – carbon monoxide, DRG – dorsal root ganglia, EC – epithelial cells, EEC – enteroendocrine cells, EGC – enteric glial cells, ENS – enteric nervous system, GIP – glucose-dependent insulinotropic polypeptide, GLP-1 – glucagon-like peptide-1, GLP-2 – glucagon-like peptide-2, IAN – intrinsic afferent neurons, ICC – interstitial Cajal cells, IGLEs – intraganglionic laminar endings, IMAs – intramuscular arrays, IN – interneurons, MN – motoneurons, NO – nitric oxide, PeNS – peripheral nervous system, PYY – peptide YY, SM – secretomotor neurons, SN – secretomotor neurons, VIP – vasoactive intestinal polypeptide

## Abstract

The digestive system has an innate monitoring and defense capacity, which allows the recognition and elimination of different dangerous substances. The complex analysis of the intestinal content comprises the cross-interactions between the epithelial cells, the enteroendocrine cells, the neural tissue and the cellular defense mechanisms. The enteric nervous system, also called "the enteric brain" or "the second brain" is the only neuronal network outside the central nervous system capable of autonomous reflex activity. The enteric nervous system activity is mostly independent of the central nervous system, but not in all aspects. In fact, even the enteral reflexes are a consequence of the bidirectional intestine-brain relation. The central nervous and enteric nervous systems are coupled through the sympathetic and parasympathetic branches of the autonomic nervous system. The gastrointestinal functions are regulated due to the interaction between the intrinsic neurons within the gastrointestinal wall and the extrinsic neurons outside the gastrointestinal tract. Here we provide an overview of the important role of the enteric brain in defensive behavior, as well as its structural and functional particularities that make it a special organ.

## INTRODUCTION

The digestive tract provides the energetic substrates and the constitutive substances needed for the proper functioning of the body. It is a unique organ being exposed to a large number of stimuli from the external environment, including microbes. The gastrointestinal surface is broad, more than 100 m^2^, compared to the skin surface, which has only 2 m^2^ [1, 2].

The digestive system also has an innate monitoring and defense capacity, which allows the recognition and elimination of dangerous chemicals, such as noxious substances produced by bacteria.

The complex intestinal content analysis comprises the cross-interactions between the epithelial cells (EC), the enteroendocrine cells (EEC), the neural tissue and the cellular defense mechanisms [3].

**Figure 1:**
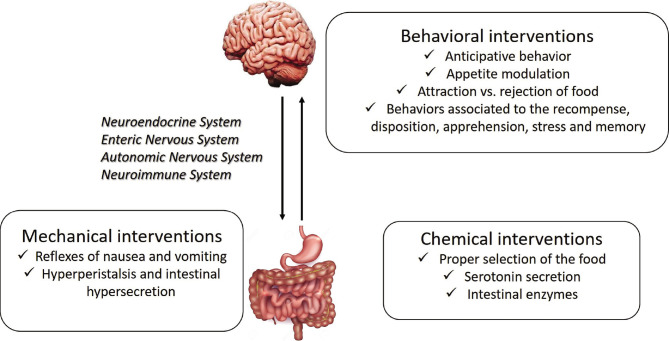
GUT-brain interaction and the main interventions involved in the response to the external factors.

The intervention of the digestive system in defense is very complex: from the elimination of the perceived harmful products, through mechanical interventions and chemical neutralization, to behavioral interventions, calling forth an anticipative-preventive behavior (Figure 1).

The digestive tract has a multi-level mechanical defense capacity: the rejection reaction appears for the first time in the oral cavity (nausea), followed by the gastric reaction (vomiting), and then the intestinal reactions (the hyperperistaltic and the intestinal hypersecretion). Besides, the unpleasant smell and the bitter taste generate aversive behaviors.

The oro-sensory system is very important for ingestive behavior and intervenes in regulating energy intake through the choice of the aliments [4]. The four tastes intervene in the generation of either positive hedonic and reward feelings of pleasure (the sweet taste) or the enhancement of the food intake (the tastes of salt, water and fat). Also, the bitter and sour tastes detect and reject dangerous or toxic food. The gustatory receptors are, in fact, chemoreceptors that are tuned to distinct classes of chemical molecules either by binding to G-protein-coupled receptors (for sweetness and umami taste) or directly to ion channel proteins (for saltiness and bitterness) [5].

The gut's place in the proper selection of food is sustained by the presence of the intestinal chemosensors, represented by specialized EEC of the gut [4]. The fact that these EEC express receptors such as T1R (for sweet taste) and T2R (for bitter taste), as well as alfa-gustducin, sustains the previous statement [6–8]. The fundamental difference between mouth and intestine, in terms of taste perception, is represented by the fact that in the oral cavity, food is undigested, while in the intestine, the absorbed nutrients generate taste signals only in specialized EEC [4].

Alpha-gustducin, involved in bitter, sweet or amino-acid taste (called umami) perception, is expressed by A, K and L cells [9, 10]. It is extensively expressed in the bowel, in less than 5% of the EC of the duodenum and none of the ordinary enterocytes [11]. In the human colon, the alpha-gustducin and peptide YY (PYY) are co-located [8]. There is no co-localization of alpha-gustducin with cholecystokinin (CCK), ghrelin or gastrin in the EEC [4]. In the mouse gut, co-localization of alpha-gustducin and serotonin was shown [8].

The taste receptors could also be expressed in pancreatic and liver cells and other sites apart from the gastrointestinal system: striated muscular fibers, myocytes, fibroblasts and central nervous system (CNS) cells [12–14]. These findings support, on the one hand, the extra gustatory functions of taste receptors and, on the other hand, that the body integrated feeding-related behavior. However, most nutrient receptors are located on EEC. EEC release hormones, which act either locally or at distance, at the level of CNS [3]. This multi-level response is an important demeanor determinant, particularly in defense responses.

Some toxic or irritant substances are detoxified at the intestinal level, and some are rejected through diarrhea and vomiting [3]. The main neurotransmitter delivered from the EEC as a consequence of the contact of the gastric or intestinal mucosa with a dangerous substance is serotonin. Serotonin has a double, central and peripheral defense action. By acting on vagal nerve endings from the gastric, duodenal or intestinal walls, it promotes the signal transmission to the area postrema, the vomiting center located in the brain [15]. Besides its central effect, serotonin initiates strong, expulsive intestinal movements, followed by emptying the undesired content [16]. Serotonin is involved in arousal, attention and particularly in avoidance behavior.

The intestinal enzymes are the first-defense line in detoxifying the foreign compounds that reach the digestive tract. The second defense line is represented by the detoxifying enzymes in the liver [3].

The digestive tract must be viewed as a very large and complex sensory system. With respect to this, the neural ways and the effectors must be investigated together. As each sensory system, the digestive tract intervenes in specific information storing as a combination between a particular compound and the related taste, the rewarding or aversive reactions. Briefly, the food receives an emotional valence (attraction *vs*. rejection). In this way, the digestive information generates anticipative behavior. Even more, the signals from the digestive tract are emotionally coded, gaining the meaning of a visceral metaphor (gut's feelings), bringing them into the consciousness field with behavioral consequences. The terms of visceral metaphors are often represented by digestive symptoms. Understanding how the enteric nervous system (ENS) mediates the behavior is an actual subject and one of great interest.

## HAS THE ENTERIC NERVOUS SYSTEM AUTONOMY?

According to Langley's studies, the ENS was viewed as a part of the peripheral nervous system (PeNS), and the intestinal neurons were framed as postganglionic parasympathetic neurons [17]. However, the ENS is the only neuronal network outside the CNS capable of autonomous reflex activity [18]. Some experimental observations, such as the coordination of intestinal peristaltic by reflexes involving the intramural nerves and the fact that the enteric neurons do not have direct contact with the parasympathetic terminations, led to the conclusion of the individuality of ENS [17].

The ENS activity is mostly independent of the CNS activity. Nevertheless, this independence must be viewed with circumspection because it refers only to certain functions. In fact, even the enteral reflexes are a consequence of the bidirectional intestine-brain relation. The CNS and the ENS are coupled through the autonomic nervous system (ANS).

Some gut functions (motility, absorption, secretion) are controlled by the ENS, while others are under CNS control. On the other hand, there are unequal influences of CNS and ENS along the digestive tract. The CNS controls the upper part of the digestive system via the brain stem and vago-vagal reflexes and the defecation via the lumbosacral spinal cord. On the contrary, gut motility is regulated by the ENS [19].

The gastrointestinal functions are regulated as a consequence of the interaction between the intrinsic neurons from the gut and the extrinsic neurons outside the gastrointestinal tract. Mechanical or chemical activation of sensory afferents is transmitted via local enteric reflexes, extraspinal CNS-independent reflexes, and CNS-dependent reflexes [3, 20].

## THE INTESTINAL BRAIN

The human ENS contains 100 million neurons, according to some authors [21], or 200–600 million, according to others [19]. The neurons are distributed in thousands of small ganglia organized mainly in two plexi, myenteric and submucosal [19].

The ENS is astonishingly similar to the brain, both in ontogeny, morpho-functional organization and chemical signaling [22]. The ENS precursors come from enteric neural crest-derived cells. These cells also delaminate from the vagal neural tube, which is the precursor of the brain and spinal cord [23, 24]. Unlike the neurons from the PeNS, collagen and Schwann cells do not support the neurons from the ENS. These neurons are sustained by enteric glial cells (EGC), similar to the astrocytes found in the CNS [25].

CNS degenerative diseases injure the ENS also. For example, in the intestinal wall of patients with Alzheimer's disease, amyloid plaques and neurofibrillary deposits have been identified [25]. Furthermore, in patients with Parkinson's disease, besides alpha-synuclein aggregation in the brain, several studies reported alpha-synuclein aggregation in the ENS [26, 27], as well as in the Lewy bodies (perikaryal-alpha-synuclein aggregates) in the ganglionic cells of enteric plexi [28].

The CNS has receptors for the neurotransmitters synthesized in ENS, and the neuromediators synthesized in the ENS are also synthesized in CNS. The complex ENS net is controlled by a large number of neurotransmitters and neuromodulators, more than in any other segment of the PeNS. This feature allows the ENS to achieve a part of its tasks independent of CNS control [25].

Bayliss and Starling have demonstrated this partial individuality of the ENS from the CNS [25]. They observed that applying pressure to the study animals' intestinal lumen produces oral contraction, anal sphincter relaxation and, in the end, a propulsive movement of the intestinal wall, defining the "law of the intestine" or the peristaltic reflex. This reflex persists even after ANS interruption, suggesting the intestine's autonomy. Trendelenburg confirmed, *in vitro*, that the peristaltic reflex could be elicited without the participation of the CNS or spinal cord [25].

Actually, the ENS is viewed as a brain due to its complex structural and functional organization. In medical literature, the ENS is often presented as "a brain in its own right", "the second brain", or "the intestinal brain" [25].

### The ENS functional organization

**Figure 2:**
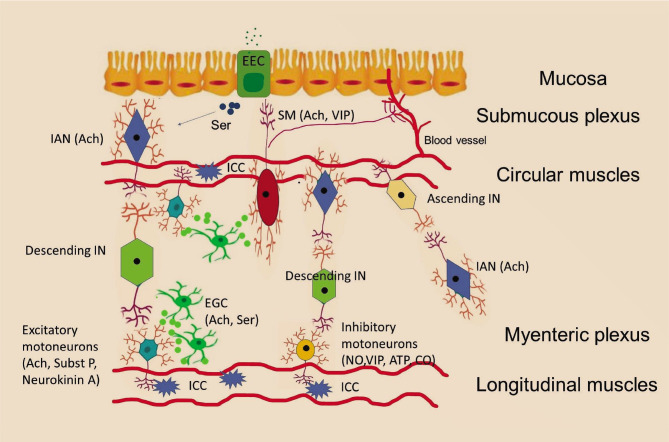
The schematic illustration of the enteric nervous system (ENS) organization. Luminal distention or distortion triggers direct activation of intrinsic afferent neurons (IAN) endings, as well as indirect activation of IAN upon serotonin (Ser) release by enterochromaffin cells (EEC) in the epithelium. IAN activate ascending and descending interneurons (IN), which stimulate excitatory and inhibitory motor neurons. The dominant neurotransmitters secreted by excitatory MN are acetylcholine (Ach), substance P (Subst P) and neurokinin A, while for the inhibitory neurons are vasoactive intestinal polypeptide (VIP), nitric oxide (NO), adenosine triphosphate (ATP) and carbon monoxide (CO). Secretomotor neurons (SM) are either cholinergic or non-cholinergic neurons that use VIP or similar peptides as principal neurotransmitters. A small group of SM sends projections to the mucosa and to the local blood vessels. The enteric glial cells (EGC) have laminar prolongations that wrap the surface of the ENS cells. The ECG and ENS cells are interconnected also via varicose nerve endings that contains a large number of vesicles. The gut contains also a special type of cells, named interstitial cells of Cajal (ICC) that are found between the nerve endings and smooth muscle cells, acting as a pace-maker.

**Figure 3:**
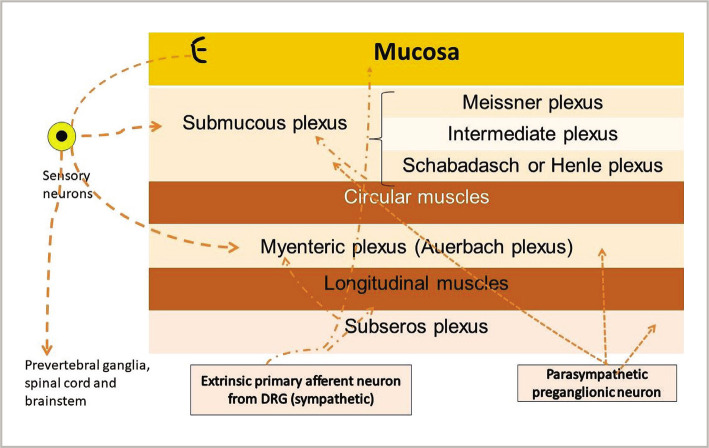
The illustration of extrinsic innervation of the gastrointestinal tract. Efferent neurons of the parasympathetic and sympathetic nervous systems synapse in the myenteric and submucosal plexuses, in the smooth muscle, and in the mucosa. DRG=dorsal root ganglia.

The ENS is located in the thickness of the gastrointestinal tract wall and is represented by interconnected webs consisting of nerve cells, EGC, EEC and interstitial cells of Cajal (ICC). These cells are gathered in small groups, named enteric ganglia. Enteric ganglia are connected to each other through nerve fiber bundles [18]. Langely described the anatomy of the ENS for the first time. He showed that the entire intestinal activity is coordinated by several types of neurons: afferent neurons, excitatory or inhibitory motoneurons (MN) and interneurons (IN).The MN have effects on a large number of cells as smooth muscle cells, endothelial cells, pacemaker cells or epithelial cells [29]. A schematic illustration of the ENS's organization is shown in Figures 2 and 3.

The ENS is mainly organized in two plexi: the myenteric (Auerbach) plexus and the submucosal plexus [18]. The myenteric plexus plays an important role in the regulation of muscle activity, while the submucous plexus is involved in the functions of the mucosa. Some neurons from these plexi have cross-functions [30].

The larger myenteric plexus is continuous along the gastrointestinal tract. It is located between the longitudinal and circular muscle layers of the gut [17]. It contains neurons involved in both motility control and the regulation of the enzyme production of adjacent organs [17]. Many myenteric neurons project into the sympathetic ganglia [31].

The submucosal plexus is a continuous layer in the intestine [19] and does not exist in the esophagus and stomach. A similar plexus is described in the gallbladder and bile ducts, but also in the pancreas [32]. The submucosal plexus has three separate layers: the Meissner plexus (the internal layer, below the muscularis mucosae), the Schabadasch or Henle plexus (the external layer, near the circular muscle layer), and an intermediate plexus (between the internal and external plexus) [33].

It has not yet been conclusively shown if the submucosal plexus neurons belong to mechanoreceptors or if their activity is triggered by the stimulation of the enterochromaffin cells, these cells being the depositaries of 95% of the serotonin found in the body [25].

To a certain extent, the myenteric and submucosal plexi are interconnected. The Meissner plexus has sensitive neurons that communicate to the ones of the myenteric plexus and motor fibers, which stimulate the epithelial crypt cells secretion [25]. Some neurons of the Henle plexus supply the innervation of the muscular layers [19] and the mucosa.

The subserous plexus is located on the digestive tract surface, as well as in the serosa of the peritoneal cavity. It is also described in the external muscular layer and is represented by fine nerve bundles in the connective tissue [19]. These fine bundles link extrinsic nerves with the ones from the deeper layers of the intestinal wall [18]. Also, neurons of the subserous plexus are in contact with the vagal branches as they penetrate the gastric and esophageal walls [18].

### The histology of ENS

The ENS consists of intrinsic afferent neurons (IAN), interneurons (IN), motoneurons (MN) and secretomotor neurons (SN) (Figure 2) [32].

The IAN form the sensory limb of all intrinsic motor and secretomotor reflexes and project circumferentially to the IN surrounding both plexuses [32]. All these neurons are cholinergic, some of them containing the substance P [32].

The IN are interposed between the IAN and the MN or SN, forming multisynaptic pathways that control the peristalsis [32].

There are two types of motoneurons: excitatory and inhibitory. The branches of the excitatory neurons are projected either nearby or rostrally to the circular muscular layer, while the branches of the inhibitory neurons are distributed caudally from the circular muscular layer. The dominant neurotransmitters secreted by excitatory MN are acetylcholine and substance P, while the inhibitory neurons are vasoactive intestinal polypeptide (VIP) and nitric oxide (NO) [32].

The EGC have laminar prolongations that wrap the surface of the ENS cells. Secondary to the release of cytokines in the gut, EGC secrete interleukins and express surface antigens (MHC class II) [32], supporting the idea that EGC modulate the inflammatory responses of the gut, apart from the control of several classical functions, like the peristalsis, blood flow and endocrine and exocrine secretions [33]. The ECG and ENS cells are also interconnected via varicose nerve endings that contain many vesicles (Figure 2) [34]. Thus, the EGC have cholinergic and serotoninergic innervation through this connection with enteric neurons [35].

The gut also contains a special type of cells called the interstitial cells of Cajal. These cells are found between the nerve endings and smooth muscle cells (Figure 2) [25]. The ICC express neither neural nor glial cell markers [25]. The ICC seems to act as a pacemaker, establishing the rhythm of bowel contractions [25].

### The enteroendocrine cells

The EEC, less than 1% of the gut cells, represent the largest endocrine organ of the human body, playing an important role in enteral information processing and transmission [36]. The EEC represent a group of specialized ECs located in the crypts and villi of the digestive tract.

The EEC communicate with the nervous system directly, chemically, and indirectly. The EEC have specialized basal processes with the role of hormones releasing and direct connection with the nerves, named neuropods [37, 38]. The information propagates through the connections between the neuropods of type I and L enterochromaffin cells and neurons, named neuroepithelial circuits [38, 39].

The direct connections between the EEC and the neurons suggest the double function of EEC, one that occurs at the top of the cell, the chemosensitizing function, and a second, conversion of this information in an electrical one at the cell base [39].

The EEC represent a very important reservoir of chemical signals, with local and remote (including CNS) effects. The EEC are stimulated by the motor fibers of the ENS and transmit information to the extrinsic afferents belonging to ANS.

The EEC respond to various substances that exist in the intestinal lumen, from nutrients to toxic products. The EEC represent the first level of intestinal information integration and the first step in the activation of neuronal networks that transmit the information to various cerebral structures [40–42]. Besides, through their actions on stomach emptying, intestinal secretion, vomiting and diarrhea, the EEC intervene in defense [43–46].

The EEC secrete a huge number of hormones that play a role in modulating appetite. These substances influence both the appetite and the metabolic control centers, as well as the pathways involved in the development of specific behaviors and memory [47].

According to the structure and location in the gastrointestinal mucosa, EEC are classified as opened and closed types. The open type EEC have a bottleneck shape and an apical luminal extension covered by microvilli. The microvilli have the role of directly detecting intestinal content. The closed type EEC do not have microvilli, do not reach the gut's content, and are found nearby the basal membrane. They are indirectly stimulated by the intestinal content via neural or humoral paths [5, 48]. Both cell types collect their secretion products into cytoplasmic small vesicles and release them by exocytosis at the baso-lateral pole after mechanical, chemical or neural stimulation. Peptides produced by EEC exert a direct effect on the adjacent nerve endings, or locally, in a paracrine manner, on other cells of the mucosa or other EEC, but also can reach distant (*e.g*., CNS) targets through their release into the bloodstream [5, 49].

The EEC release more than 30 peptides, most regulating the physiological processes related to digestion. The balance between orexigenic and satiety signals is particularly important in the early phase of digestion when the orexigenic signals are decreasing and the satiety rising, as well as at the start of the interdigestive phase when this situation is reversed [50].

The most studied peptides produced by EEC are motilin and ghrelin, released through the interdigestive time and cholecystokinin (CCK), glucose-dependent insulinotropic polypeptide (GIP), glucagon-like peptide- 1 (GLP-1) and peptide YY (PYY) released after food intake [51]. The most important peptides released by the enteroendocrine cells and their main roles are described in Table 1 [52–77].

**Table 1:** The most important peptides released by the enteroendocrine cells.

Peptide	Place of secretion	Time of secretion	Effect
**Motilin [51]**	M-cells of the duodenum and jejunum	Cyclically released during fasting period	Controls the inter-digestive contractions of the gut; Stimulates gastric activity Orexigenic hormone.
**Ghrelin [52-54]**	A-cells of the stomach	Produced in pre-prandial period and its level peaks prior the food intake and rapidly decreases when gastric content is evacuated into the duodenum	Orexigenic hormone
**Glucose-dependent insulinotropic polypeptide (GIP) [51, 52]**	K-cells of the intestinal mucosa	After food ingestion	Stimulates postprandial insulin secretion (25-70%); Promotion of growth and survival of the pancreatic beta-cell.
**Glucagon-like peptide- 1 (GLP-1) [55-63]**	L-cells of the intestinal mucosa	After food ingestion in association with PYY	Anorexigenic peptides; Inhibits gastric emptying and gastric secretion; Ileal brake; Stimulates insulin secretion.
**Peptide YY (PYY) [55, 58-65]**	L-cells of the intestinal mucosa	After food ingestion	Anorexigenic peptides; Inhibits propulsive activity in the proximal segment of intestine; Ileal brake; Food aversion and reduction of energy intake (peptide YY3-36).
**Glucagon-like peptide-2 (GLP-2) [66-68]**	L-cells of the intestinal mucosa	After food ingestion	Intestinal lipid absorption; Delay gastric emptying; Promotes intestinal epithelial growth; Role in the defence of the mucosa.
**Cholecystokinin (CCK) [69, 70]**	I-cells of the intestinal mucosa	Its secretion is triggered by the presence of food in the intestine, mainly after meals rich in fat and protein	Anorexigenic hormone
**Oxyntomodulin (OXM) [70–72]**	L-cells of the intestinal mucosa	Post-prandial state	Anorexigenic peptides; Direct effects on the hypothalamus; Increases energy expenditure; Delay gastric emptying; Stimulates insulin secretion.
**Serotonin [73-75]**	K-cell of the proximal gut	After food ingestion	Promoting the intestinal motility; Intestinal secretion regulation; Role in CNS signaling.
**Leptin [76, 77]**	P-cells	During feeding or overfeeding	Neurohormone that stimulates the discharging of afferent vagal neurons; Potentiates the CCK effects on satiety.

### The intrinsic ENS

#### Motor (effector) intestinal neurons (MN)

The enteric MN are either excitatory or inhibitory to gut muscle [78]. The excitatory MN synthesize choline-acetyltransferase and tachykinins, especially substance P and neurokinin A [79]. The main neurotransmitter is acetylcholine, the role of tachykinins being only a minor one [80]. The inhibitory MN has NO-synthase and produce nitric oxide (NO) [81]. Beside NO, these neurons release the vasoactive intestinal polypeptide (VIP) [82], adenosine triphosphate (ATP) [83] and carbon monoxide (CO) [84].

#### Secretomotor neurons (SM)

In the myenteric plexus, there are two types of SM: cholinergic neurons and non-cholinergic neurons that use VIP or similar peptides as principal neurotransmitters. A small group of cholinergic neurons sends projections to the mucosa and the local blood vessels [29]. The non-cholinergic neurons intermediate most of the local reflex responses [85, 86].

The intrinsic afferent neurons (IAN) are also named intrinsic primary afferent neurons or primary enteric afferent neurons. IAN could be considered a distinct class because they have characteristic electrophysiological properties. These types of neurons are present in both plexi [29], representing about 30% of myenteric neurons and 14% of submucosal neurons [87]. Some terminations of the IAN in the mucosa may have secretomotor effects [78].

The interneurons are ascending (rostrally directed) and descending (caudally directed). The IN appear crucial in controlling and coordinating intestinal activity. Distinct from the MN that have very short projections, less than 16 mm, the IN project up to 68 mm [88].

There are three types of descending IN: ChAT/NOS/VIP positive (regulates the local motility reflexes), ChAT/SOM positive (which has a role in the transport of migrating myoelectric complexes) and ChAT/5-HT positive (regulates the secretomotor functions) [78, 89].

### The extrinsic digestive tract innervation

The direct innervation of the digestive tract is realized by parasympathetic and noradrenergic endings (Figure 3). Parasympathetic innervation is supplied by the vagus nerve and the pelvic nerve. The vagal innervation is directed to the upper gastrointestinal tract, including the striated muscle of the upper third of the esophagus, the wall of the stomach, the small intestine, and the ascending colon. The pelvic nerve innervates the lower gastrointestinal tract, including the striated muscle of the external anal canal and the walls of the transverse, descending, and sigmoid colons. The noradrenergic terminations are directed to the smooth muscle, mainly the circular fibers of the sphincters, and to the arteries within the gut wall, producing spasm and vasoconstriction [89].

There are five types of extrinsic sensory neurons: neurons with intraganglionic laminar endings (IGLEs), mucosal afferents, muscular–mucosal afferents, intramuscular arrays (IMAs), and vascular afferents [90].

IGLEs are found in the capsule of ganglia from the myenteric plexus [91]. They are mechanoreceptors with a low activation threshold and respond to passive or active muscle tension [92]. There is bidirectional communication between the IGLEs and the ENS neurons [93]. The signals from the IGLEs generate the vagal afferent tone, which contributes to balanced interoceptive awareness and emotional well-being [94]:

- IMAs are mechanoreceptors activated by stretch [4]. They are almost exclusively located in gastric muscular layers [95, 96]. IMAs form synapse-like complexes with ICC [97];
- Muscular–mucosal afferents are activated by mucosal stroking or stretch [98].

The mucosal afferents do not go through the epithelial layer, and they are indirectly activated by the signals released by the epithelial cell, mainly by the EEC [99]. The fibers that arise from dorsal root ganglia (DRG), as well as their sensory terminations projected around the mesenteric arteries are sensitive to direct mechanical stimulation [90]. The majority of extrinsic afferent neurons from the bowel are not sensitive to the environment, so-called silent nociceptors, being musculo-mucosal afferents or vascular afferents [90].

There are four extrinsic sensory pathways: the vagal sensory innervation, the sensory innervation provided by the thoracolumbar and lumbosacral dorsal root ganglia, as well as viscero-fugal pathways [91].

The vagal branches transmit information related to the physiological status of the gut, while the sympathetic thoracolumbar tracts convey information related to pain and gastrointestinal discomfort [91].

## CONCLUSION

The concept of "enteric brain" is developed in relation with the description of complex functions such as gut defense, anticipative-defense behavior or visceral metaphors. The enteric nervous system relies on the same nervous cell type and neurotransmitters/neuromodulators found in the central nervous system, being named by some authors "the second brain". This "second brain", in communication with the central nervous system, is involved in the etiopathogenesis of certain diseases, the control of interoceptive awareness and emotional well-being, and the behaviors associated with recompense, disposition, apprehension, stress and memory.
